# Elastic Modulus of the Alkali-Silica Reaction Rim in a Simplified Calcium-Alkali-Silicate System Determined by Nano-Indentation

**DOI:** 10.3390/ma9090787

**Published:** 2016-09-20

**Authors:** Kunpeng Zheng, Mladena Lukovic, Geert De Schutter, Guang Ye, Luc Taerwe

**Affiliations:** 1 Magnel Laboratory for Concrete Research, Department of Structural Engineering, Ghent University, Ghent 9052, Belgium; geert.deschutter@ugent.be (G.D.S.); G.YE@TUDELFT.NL (G.Y.); Luc.Taerwe@UGent.be (L.T.); 2 Microlab, Faculty of Civil Engineering and Geosciences, Delft University of Technology, Delft 2628 CN, The Netherlands; M.Lukovic@tudelft.nl

**Keywords:** alkali-silica reaction, reaction rim, calcium alkali silicate, elastic modulus

## Abstract

This work aims at providing a better understanding of the mechanical properties of the reaction rim in the alkali-silica reaction. The elastic modulus of the calcium alkali silicate constituting the reaction rim, which is formed at the interface between alkali silicate and Ca(OH)_2_ in a chemically-idealized system of the alkali-silica reaction, was studied using nano-indentation. In addition, the corresponding calcium to silica mole ratio of the calcium alkali silicate was investigated. The results show that the elastic modulus of the calcium alkali silicate formed at the interface increased with the increase of the calcium to silica mole ratio and vice versa. Furthermore, the more calcium that was available for interaction with alkali silicate to form calcium alkali silicate, the higher the calcium to silica mole ratio and, consequently, the higher the elastic modulus of the formed calcium alkali silicate. This work provides illustrative evidence from a mechanical point of view on how the occurrence of cracks due to the alkali-silica reaction (ASR) is linked to the formation of the reaction rim. It has to be highlighted, however, that the simplified calcium-alkali-silicate system in this study is far from the real condition in concrete.

## 1. Introduction

The alkali-silica reaction (ASR) is a common durability problem affecting concrete structures. It contains a series of chemical and physical interactions between the reactive silica in the aggregates and the pore solution of the cement paste resulting in a slow, but severe expansive deterioration of the concrete. Although ASR has been studied for several decades since it was firstly spotted in the 1940s, the mechanism of the generation and accumulation of the interior force at the micro scale causing the expansion and cracking of a concrete structure at the macro scale is still unclear.

**Figure 1:**
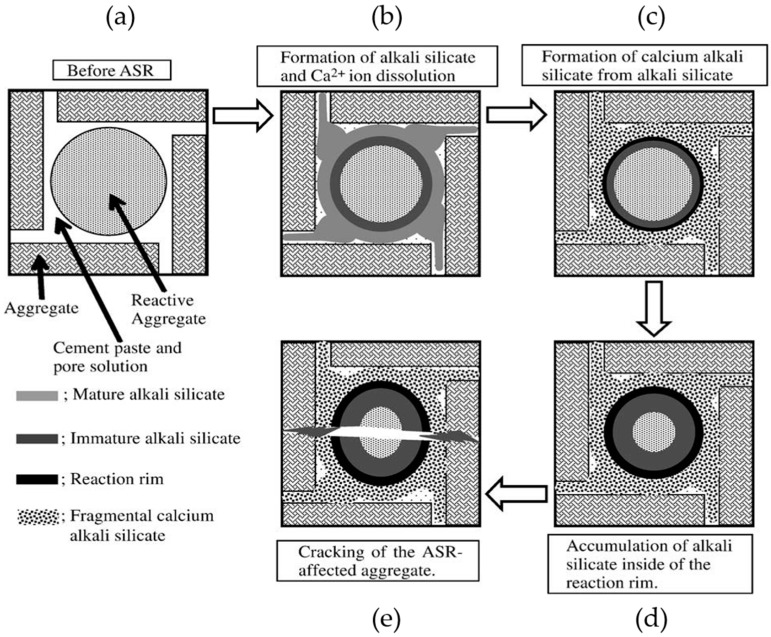
Schematic representation of the mechanism of the alkali-silica reaction (ASR) in concrete, modified from [7]. (**a**) before ASR; (**b**) formation of alkali silicate; (**c**) formation of calcium alkali silicate from alkali silicate; (**d**) accumulation of alkali silicate inside of the reaction rim; (**e**) cracking of the ASR-affected aggregate.

Regarding this issue, many theories and models have been proposed [1,2,3,4,5]. The most recent one is the reaction rim theory from Ichikawa et al. in 2007 [6], linking the uncommon occurrence of the reaction rim in the ASR-affected concrete with the generation and accumulation of the interior expansive force. According to this model [6,7], which is schematically shown in Figure 1, the reaction rim develops by the formation and accumulation of calcium alkali silicate, which forms due to the interaction between alkali silicate and Ca^2+^ at the interface between the aggregate and the cement paste. Once the reactive silica particle is completely covered by the reaction rim, the reaction rim is able to behave as a semi-permeable membrane to prevent the extrusion of alkali silicate out of the reaction site while allowing the penetration of alkaline solution to attack the reactive silica and generate alkali silicate continuously; see Figure 1a–c. As a result, alkali silicate will accumulate within the reaction rim, applying an expansive force on the rim; see Figure 1d. In response, the reaction rim exerts a constraint on this region until the expansive force exceeds the strength of the rim and the rim is broken; see Figure 1e.

**Figure 2:**
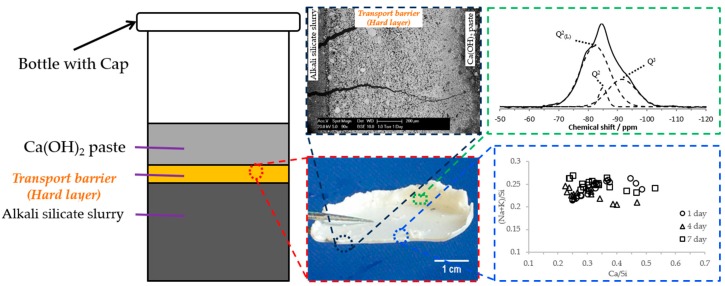
Graphical summary of our previous study [8].

However, how the reaction rim is formed from the interaction between alkali silicate and calcium and why it is able to act as a semi-permeable membrane to prevent the penetration of alkali silicate while allowing the penetration of alkaline solution is not clear from a fundamental chemical point of view. In addition, it is not known how much constraint the reaction rim can provide before its breakage due to the expansive force generated by the accumulation of alkali silicate. The prior work of our study [8] focused on the former; the present work focuses on the latter. Considering the importance of our prior work to the present study, a brief introduction is shown in Figure 2 and illustrated as follows.

In our prior study [8], the existence of a transport barrier similar to the reaction rim was demonstrated. The transport barrier was also called the hard layer based on the observation. As shown in Figure 2, the transport barrier with a thickness of around 1 mm was located at the interface between the alkali silicate slurry and the Ca(OH)_2_ paste in a calcium-alkali-silicate system, which simulates the formation of the reaction rim around the reactive aggregate in concrete. Comparable to the reaction rim proposed by Ichikawa et al. [6], this transport barrier was able to prevent the transport of calcium and silicate, separate the system into layers and allow the penetration of alkalis. According to the elemental composition analysis, this transport barrier was composed of calcium alkali silicate with the calcium to silica mole (Ca/Si) ratio ranging from 0.22–0.53 and the alkali to silica mole ((Na + K)/Si) ratio ranging from 0.20–0.26. Moreover, from a nano-structural point of view regarding the silicon-oxygen networks of the calcium alkali silicate constituting the transport barrier, the presence of Q^2^ and Q^3^ polymerization indicated its chain-like structure with interlinkages according to the ^29^Si nuclear magnetic resonance (NMR) results. Therefore, it is rational to believe that the formation and evolution of the reaction rim proposed by Ichikawa et al. [6] was realized in our chemical model system. Notably, although the hard layer found in our prior work was acting as a transport barrier, which had a similar role as the reaction rim, we will keep addressing it as the hard layer or the transport barrier in this study, since we investigated a chemical model and not concrete.

According to Ichikawa [6,7], besides acting as a semi-permeable membrane, the reaction rim should be able to apply a mechanical constraint in response to the expansive force generated by the accumulation of alkali silicate before its breakage. The elastic modulus, as one of the key factors needed to evaluate the behavior of a material under stress, is of great relevance in linking the mechanical performance of the reaction rim under the expansive force due to ASR with the intrinsic properties of the calcium alkali silicate constituting the reaction rim. In light of this consideration, the elastic modulus of the calcium alkali silicate constituting the transport barrier formed at the interface of alkali silicate with calcium was investigated with nano-indentation in this work. Thereafter, the relationship between the elastic modulus of the calcium alkali silicate and its elemental composition was studied.

## 2. Materials and Methods

### 2.1. Materials

**Figure 3:**
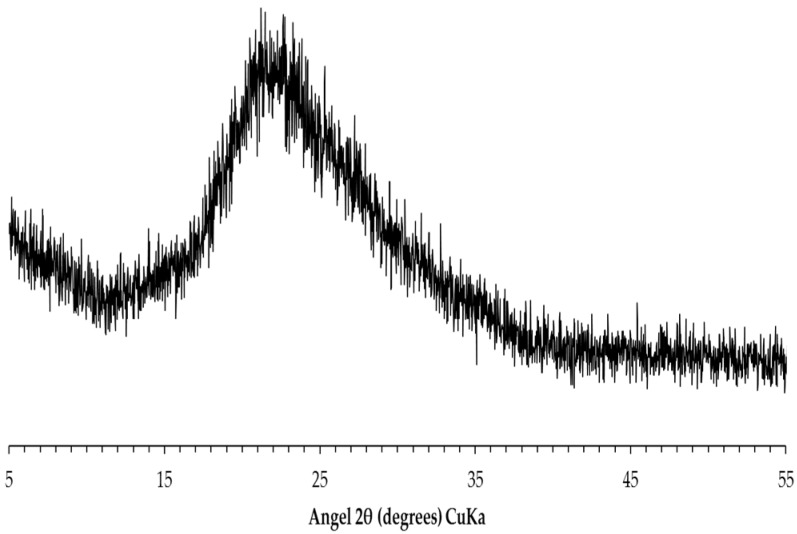
XRD pattern of silica fume used in this study.

In order to simulate the interaction between the reactive silica in the aggregates and the pore solution of the cement paste with an accelerated reaction rate, silica fume (Elekem Microsilica Grade 940U, Oslo, Norway) was chosen as the silica source in this study. The composition of the used silica fume in the form of oxides is given in Table 1, and the X-ray diffraction (XRD) pattern of the used silica fume is shown in Figure 3. To simulate the pore solution of the cement paste, the NaOH solution with a concentration of 1 mol/L was obtained by dissolving pellets of NaOH in deionized and CO_2_-free water. The Ca(OH)_2_ paste was used for simulating the calcium source in concrete, where it is present both as cations (Ca^2+^) in the pore solution and as free portlandite (Ca(OH)_2_) predominantly near the aggregate surface [9]. The Ca(OH)_2_ paste was obtained by mixing a certain amount of Ca(OH)_2_ powder with 1 mol/L NaOH solution at a liquid to solid mass ratio of 3 to maintain a constant liquid to solid ratio and the pH level of the Ca(OH)_2_ paste with the alkali silicate slurry before and after the addition of the Ca(OH)_2_ paste.

### 2.2. Sample Preparation

**Figure 4:**
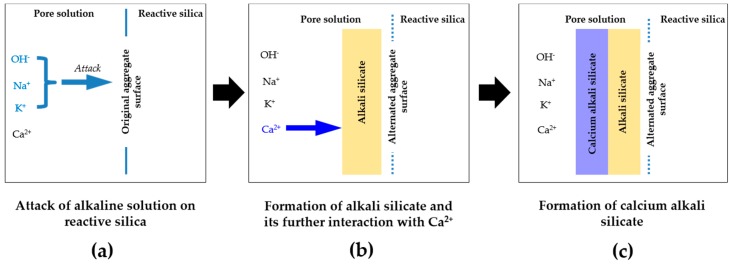
Schematic representation of the interaction of reactive silica with the constituents in the pore solution of cement paste: (**a**) attack of alkaline solution on reactive silica; (**b**) formation of alkali silicate and its further interaction with Ca^2+^; (**c**) formation of calcium alkali silicate.

To simulate the first two steps of the formation of the reaction rim as shown in Figure 4a,b, the alkali silicate slurry as the product of this process was obtained from the interaction between the silica fume and 1 mol/L NaOH solution. The detailed process is given as follows: (1) 1 mol/L NaOH solution was added to 20 g of silica fume at a liquid to solid mass ratio of 3 to simulate partial silica dissolution in ASR [10]; (2) the mixture was sealed in a polypropylene bottle and rotated using a rotary mixer at a speed of 60 rpm for 24 h at room temperature; (3) the mixture was taken off the mixer and left at rest for another 24 h for stratification. These steps simulated the situation near the surface of the reactive silica particle where unreacted silica, dissolved and undissolved alkali silicate co-exist, as shown in Figure 4b.

Afterwards, the Ca(OH)_2_ paste was added gently to the top of the alkali silicate slurry without shaking or stirring to avoid disturbance. Subsequently, the mixture was sealed again and left at rest for another 7 days for further evolution. This is enough for the system to reach the equilibrium according to our previous studies. This step simulated the contact of the calcium present in the form of both Ca^2+^ and free Ca(OH)_2_ with the dissolved and undissolved alkali silicate at the interface between the alkali silicate and calcium near an aggregate surface, as shown in Figure 4b. The amount of the Ca(OH)_2_ paste added to the system was set to 1 g, 5 g and 10 g to simulate the different situations near the aggregate surface during the formation of the reaction rim in the sense that different amounts of calcium may be available for its interaction with alkali silicate. Notably, the availability of calcium near the aggregate surface is associated with the distance to the aggregate surface: more calcium is present close to the cement paste, while less calcium is present close to the aggregate. With this consideration, three systems created in this study are introduced as follows:

(1) The system under the condition that the amount of calcium is extremely low due to the consumption by its interaction with alkali silicate at an earlier time [10]. In this study, this shortage of calcium results in the formation of calcium alkali silicate, which is not sufficient to completely cover the surface of the alkali silicate slurry.
(2) The system under the condition that a moderate amount of calcium leads to a complete coverage of the surface of the alkali silicate slurry in this study.
(3) The system under the condition that an excess amount of Ca(OH)_2_ is present when the surface of the alkali silicate slurry is completely covered in this study. This situation usually happens at the location close to the pore solution of the cement paste where abundant Ca(OH)_2_ is present.

**Figure 5:**
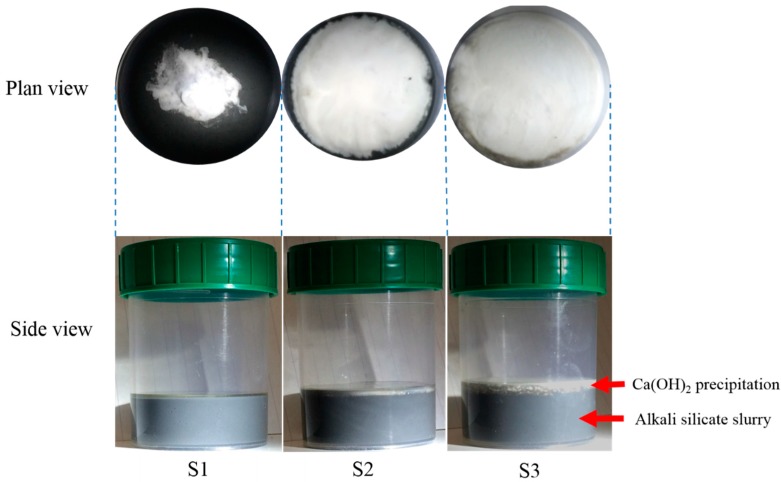
The appearance of the mixture after the Ca(OH)_2_ paste addition of 1 g, 5 g and 10 g in S1, S2 and S3, respectively.

These systems, after the addition of the Ca(OH)_2_ paste, are shown in Figure 5 and illustrated as follows:

(1) For the sample with a Ca(OH)_2_ paste addition of 1 g (S1), the added portlandite precipitated at the top surface of the alkali silicate slurry without covering its whole surface, enabling the free transport of alkali silicate from the uncovered surface;
(2) For the sample with a Ca(OH)_2_ paste addition of 5 g (S2), the added Ca(OH)_2_ precipitated and just covered the whole surface of the alkali silicate slurry;
(3) For the sample with a Ca(OH)_2_ paste addition of 10 g (S3), the added Ca(OH)_2_ not only covered the surface of the alkali silicate slurry, but also precipitated more on top of it; the thickness of the sediment is about 2–3 mm.

**Figure 6:**
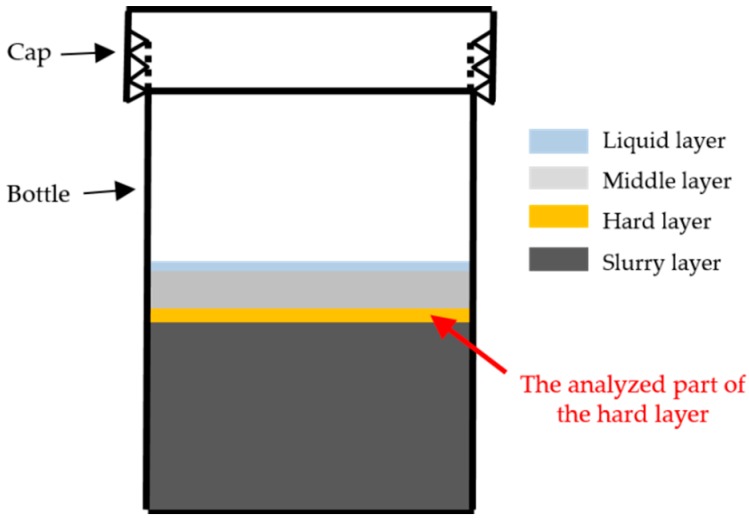
Schematic diagram of the layered system.

**Figure 7:**
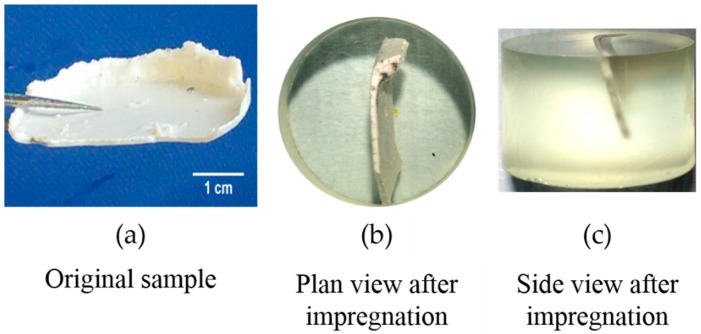
Sample before and after impregnation. (**a**) Sheet-like sample collected from the hard layer; (**b**) plan view of the sheet-like sample after impregnation; (**c**) side view of the sheet-like sample after impregnation.

After the 7 days of storage, samples from the different systems were collected. According to the observation during the sample collection, the appearance of the system is schematically shown in Figure 6. From top to bottom, there were four layers in this system: (1) the liquid layer at the top; (2) the middle layer where excess Ca(OH)_2_ precipitated (only for S3); and (3) the hard layer separating the precipitated Ca(OH)_2_ from (4) the alkali silicate slurry layer. The location of the samples collected for the investigation in this study is marked in Figure 6, and their appearance is shown in Figure 7a.

As shown in Figure 7a, the hard layer was collected from three systems and immediately immersed in isopropanol for 7 days to remove the water and, therefore, to stop any reaction. Isopropanol was renewed every day. Meanwhile, the adhesives on the surface of the samples were carefully removed with a brush during the isopropanol immersion. After 7 days of immersion, the samples were transferred to a desiccator with silica gel and portlandite and left there for 2 weeks to dry. Afterwards, the samples were impregnated vertically in an epoxy to expose the surface of their cross-section, as shown in Figure 7b,c. After the impregnation (wet potting to avoid the damage from the vacuum) and polishing by following the procedures proposed by Stutzman [11], the samples were ready for analysis.

### 2.3. Nano-Indentation Testing

Nano-indentation is a micromechanical test. Its principle and measuring procedure are similar to the hardness test, but at a micro scale. With the help of nano-indentation, local mechanical properties including the elastic modulus and hardness of the tested points can be determined [12]. However, it has to be emphasized that the obtained measurement of nano-indentation is highly sensitive to the surface roughness of the tested surface [13]. As a consequence, micromechanical properties obtained by various researchers have a wide variation range and, therefore, should be critically analyzed.

In this study, the Agilent Nano Indenter G200 (Agilent Technologies, Santa Clara, CA, USA) was used for nano-indentation [14,15] to investigate the elastic modulus of the cross-section of the hard layer, which was located at the interface of the alkali silicate slurry and the Ca(OH)_2_ paste. A Berkovich tip was used as the indenter of nano-indentation, and both the loading and unloading rate were set at 0.001 mN/s. The indentation depth was 2000 nm. Particularly, the grid for S1 with a Ca(OH)_2_ paste addition of 1 g was set to consist of 5 rows with 15 indents per row; the grid for S2 with a Ca(OH)_2_ paste addition of 5 g was set to consist of 3 rows with 20 indents per row; the grid for S3 with a Ca(OH)_2_ paste addition of 10 g was set to consist of 7 rows with 10 indents per row. The distance between neighboring indents was 40 μm. The continuous stiffness method (CSM) was applied for testing [16].

### 2.4. Scanning Electron Microscope Imaging and Elemental Composition Analysis

Back-scattered electron (BSE) and secondary electron (SE) images of the indented region of each sample were obtained by using scanning electron microscope (SEM) JEOL JSM 5600 (JEOL, Tokyo, Japan). The BSE images were used for qualitatively checking the capillary porosity of the samples. During the elemental composition analysis with the energy-dispersive X-ray spectroscopy (EDS) within the SEM, the SE images were used for locating the indentation imprint. The area-scanning of EDS analysis was carried out on each indentation imprint. The size of the area of each indentation imprint selected for EDS analysis was set to be the largest indentation imprint of all of the indentations on each sample to avoid errors introduced by the different sizes of the indentation imprint.

## 3. Results and Discussion

### 3.1. Nano-Indentation Measurements

**Figure 8:**
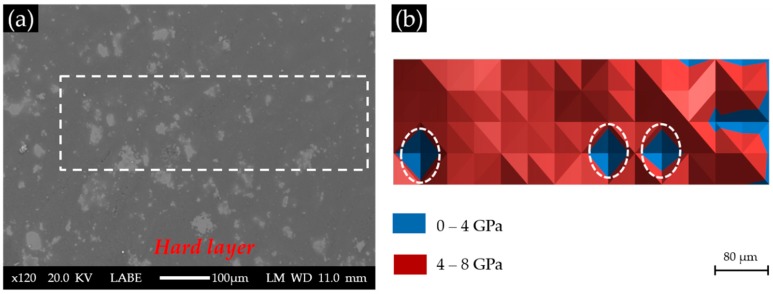
(**a**) The back-scattered electron (BSE) image of S1 with a Ca(OH)_2_ paste addition of 1 g; (**b**) the corresponding elastic modulus.

**Figure 9:**
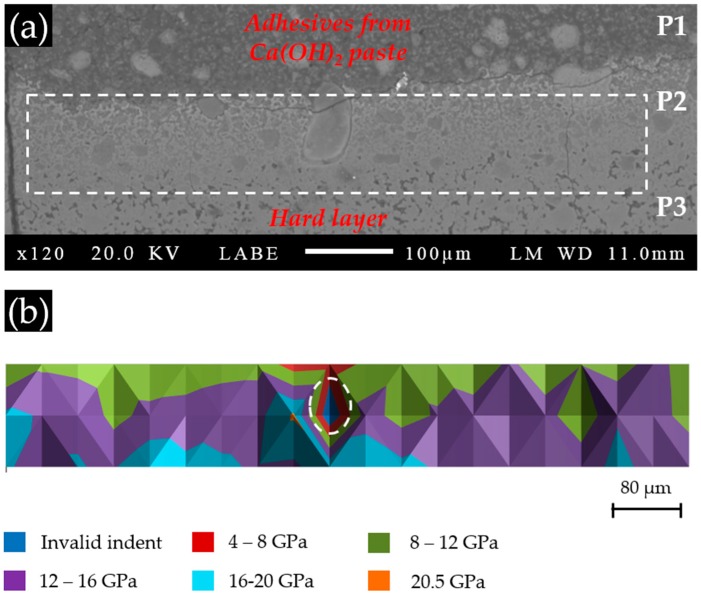
(**a**) The BSE image of S2 with a Ca(OH)_2_ paste addition of 5 g; (**b**) the corresponding elastic modulus.

**Figure 10:**
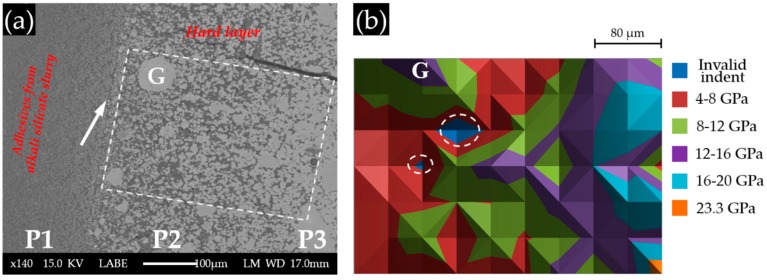
(**a**) The BSE image of S3 with a Ca(OH)_2_ paste addition of 10 g; (**b**) the corresponding elastic modulus. P1, Part 1.

Areas where the indentations were performed are marked with a rectangle in the BSE images of each sample, as shown in Figure 8a, Figure 9a and Figure 10a for S1, S2 and S3, respectively. The elastic modulus of each area measured by nano-indentation was plotted according to its position (X–Y) to give an elastic modulus map, as shown in Figure 8b, Figure 9b and Figure 10b for S1, S2 and S3, respectively.

According to the BSE image of Sample S1 with a Ca(OH)_2_ paste addition of 1 g, as shown in Figure 8a, the surface of the cross-section of the hard layer was generally uniform except for some areas with a higher grey scale level. These brighter areas were thought to have more products that reflect the electron beams from the SEM. As shown in Figure 8b, most of the indented areas of this sample had elastic moduli ranging from 4 to 8 GPa, while several areas located on the right had elastic moduli below 4 GPa. Notably, the three areas marked with circles are the invalid indents due to surface defects. Apparently, the elastic modulus of the areas composed of the calcium alkali silicate found in this sample (S1) was much lower than those reported in the literature ranging from about 20 GPa to about 30 GPa [17]. This was probably because too little calcium was available in the system to form a stiffer calcium alkali silicate, since a higher calcium content usually leads to the formation of a calcium alkali silicate with a higher elastic modulus [18]. This calcium alkali silicate with low calcium content may form directly by the interaction of alkali silicate with calcium or by the further interaction of alkali silicate with existing calcium alkali silicate [19], given the abundant presence and free transport of alkali silicate through the uncovered surface in S1.

As shown in Figure 9a, the whole region of the hard layer in Specimen S2 can be divided into three parts according to their different grey scale levels: (1) the dark part at the top (P1), where rare product was available to reflect electron beams: this part was located at the region of the Ca(OH)_2_ paste attached to the hard layer; (2) the intermediate part (P2), which was located at the transition zone of the bright part (the hard layer) with the dark part (the adhesives); (3) the bright part, completely located at the hard layer, at the bottom (P3), where large amounts of calcium alkali silicate were present. Therefore, combining with the location of the hard layer shown in Figure 6, P1 was from the Ca(OH)_2_ paste; P2 was located at surface of the hard layer in contact with the Ca(OH)_2_ paste; P3 was completely located at the hard layer closer to the slurry layer than the others. The grid of indentations on this sample is indicated with the rectangle in Figure 9a.

The nano-indentation results of the areas in Sample S2 are shown in Figure 9b. The first row of indents, located near the border between the hard layer and the region of adhesive, had low elastic moduli ranging from 8 to 12 GPa, indicating that the calcium alkali silicate present at this place was either weak or rare. The second row had, in general, higher values of elastic moduli compared to the first row, ranging from 12 to 16 GPa. This was possibly caused by a more densified structure present in the second row. The third row had slightly higher values of elastic moduli ranging from 12 to 20 GPa. This was probably caused by the formation of a stiffer calcium alkali silicate at this location, since this region appeared to be as equally densified as the second row according to their similar grey scale levels from the BSE images. The area marked with a circle corresponds to an invalid indent.

The BSE image of Sample S3 with a Ca(OH)_2_ paste addition of 10 g is shown in Figure 10a. Obviously, three parts can be differentiated according to their grey scale level. (1) P1 has the lowest grey scale level, suggesting that it is porous. Furthermore, the large difference in appearance between P1 and the other parts, as well as the observation during sample collection indicates that P1 was located at the region of the adhesive from the alkali silicate slurry. Its border with the hard layer is marked with an arrow in Figure 10a; (2) P2 was located at the hard layer. An increasing amount of calcium alkali silicate appeared as groups constituting a less porous, but disorganized structure in this region. This disorganized appearance of calcium alkali silicate in groups (the largest is indicated in Figure 10a by “G”), as the product of the interaction between alkali silicate and calcium, confirmed our hypothesis that neither alkali silicate nor calcium was well dispersed at their interface given that no shaking or stirring was applied to the mixture; (3) P3 was located at the region of the hard layer, further away from the border of the hard layer and the adhesives than the other two parts. Obviously, P3 contained the most calcium alkali silicate among these three parts leading to the formation of a more densified structure. Unlike the appearance of P2, the calcium alkali silicate in P3 was present as a continuous phase rather than as groups, implying that the availability of alkali silicate and calcium was sufficient enough to ensure the development of such a continuous phase of calcium alkali silicate. Pores were still visible in P3, but with a decreased amount and size compared to P2. Interestingly, the groups of calcium alkali silicate in P2, particularly the one marked with “G” in Figure 10a, had a comparable grey scale level to that of the continues phase in P3. This implies that the porosity of the areas from P2 and P3 were almost the same. Therefore, combining with the location of the hard layer given in Figure 6, P1 was located at the adhesives from the slurry layer; P2 was located at the hard layer in contact with the alkali silicate slurry; P3 was located at the hard layer and further away from the alkali silicate slurry than the others.

The nano-indentation results of Sample S3 with a Ca(OH)_2_ paste addition of 10 g are shown in Figure 10b. The grid of indents were located within P2 and P3. Based on the results shown in Figure 10b, three layers can be differentiated from left to right according to their variation in elastic moduli. (1) The first layer from the left, located within the region of P2, had values of elastic moduli ranging from 4 to 8 GPa. As shown in the BSE image, the region where the first layer was located had a porous structure resulting in low values of the elastic moduli. However, the group of calcium alkali silicate marked with “G” had a higher elastic modulus ranging from 12 to 16 GPa than its neighbors in P2; (2) The second layer from the left with the values of elastic moduli ranging from 8 to 12 GPa was located at an intermediate place between P2 and P3, where more calcium alkali silicate was present and less pores were found; (3) The third layer was fully within the territory of P3, which was the most densified region of this sample, having the values of elastic moduli ranging from 12 to 20 GPa. The two areas marked with circles are the invalid indents.

This distribution of the elastic moduli suggested the possible relationship between elastic modulus and the grey scale level and the local porosity. Indeed, the higher the grey scale level, the lower the local porosity of the indented areas, resulting in a higher value of the elastic modulus. In other words, it seems that the local porosity dominantly determined the elastic moduli of the areas composed of calcium alkali silicate in this sample.

### 3.2. Elemental Composition Analysis

**Figure 11:**
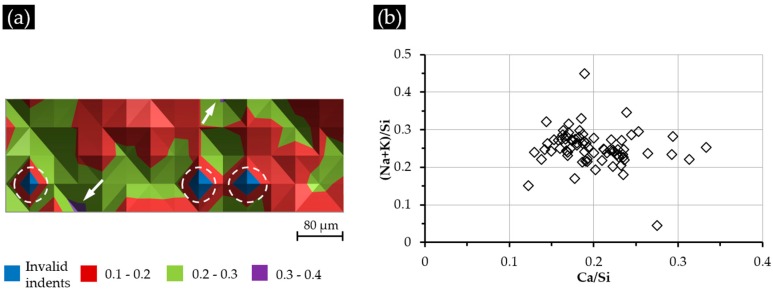
(**a**) The Ca/Si ratio variation of the indented areas from S1; (**b**) the elemental composition of the indented areas.

**Figure 12:**
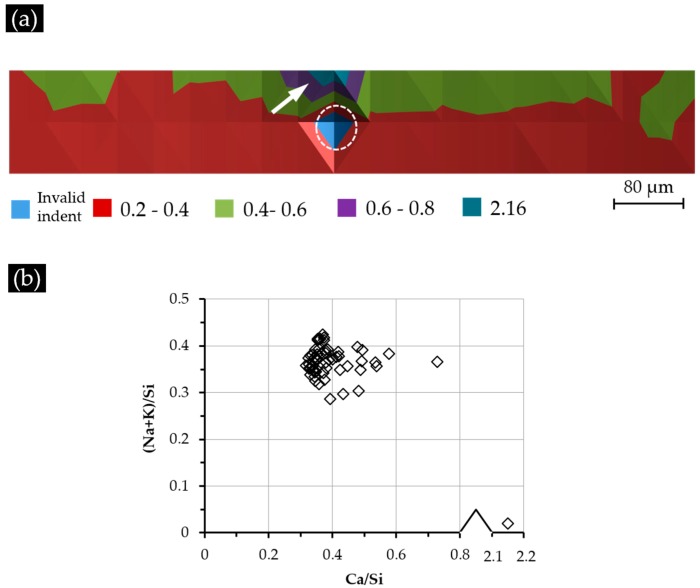
(**a**) The Ca/Si ratio variation of the indented areas from S2; (**b**) the elemental composition of the indented areas.

**Figure 13:**
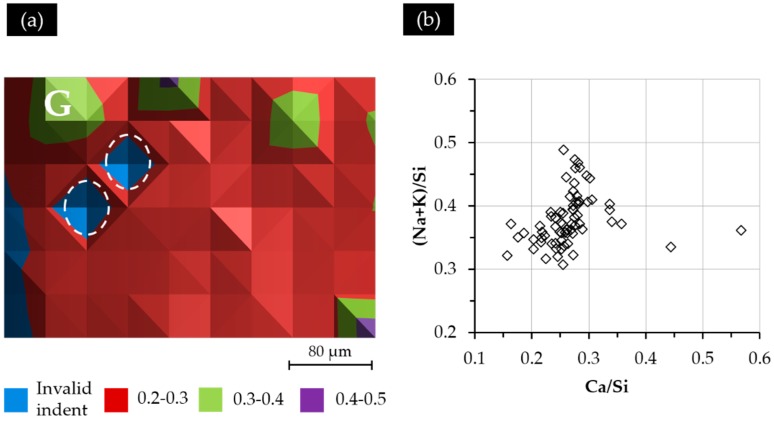
(**a**) The Ca/Si ratio variation of the indented areas from S3; (**b**) the elemental composition of the indented areas.

The elemental composition of each indented area measured by EDS was expressed as the calcium to silica mole (Ca/Si) ratio and the alkali to silica mole ((Na + K)/Si) ratio. Thereafter, the Ca/Si ratios of each indented area were plotted against its position (X–Y):

(a) Figure 11a for S1 (with a Ca(OH)_2_ paste addition of 1 g);
(b) Figure 12a for S2 (with a Ca(OH)_2_ paste addition of 5 g);
(c) Figure 13a for S3 (with a Ca(OH)_2_ paste addition of 10 g).

Besides, the Ca/Si ratio and (Na + K)/Si ratio of all of the indented areas are plotted in Figure 11b, Figure 12b and Figure 13b to reveal the elemental composition of the calcium alkali silicate from S1, S2 and S3, respectively.

The Ca/Si ratio variation of the indented areas of Sample S1 is shown in Figure 11a. Most of the indented areas show a Ca/Si ratio of less than 0.30, except two, which had a higher Ca/Si ratio ranging from 0.30–0.40 and whose locations are indicated with arrows in Figure 11a. Regardless of these two areas, nearly half of the areas had a Ca/Si ratio ranging from 0.10–0.20, and the others had a Ca/Si ratio ranging from 0.20–0.30. The locations of three invalid indents are marked with three circles; the elemental compositions of these areas were excluded from the results accordingly. Similar to its corresponding nano-indentation results given in Figure 8, the distribution of the Ca/Si ratio does not show any typical pattern indicating a generally homogeneous distribution of calcium and silicon in the hard layer of S1.

As shown in Figure 11b, the indented areas of S1 had the Ca/Si ratio ranging from 0.12–0.33 and the (Na + K)/Si ratio ranging from 0.05–0.45. Moreover, most of the areas (81%) had the Ca/Si ranging from 0.15–0.25, and 86% of the indented areas had the (Na + K)/Si ratio ranging from 0.20–0.30. This elemental composition of the calcium alkali silicate was comparable to the ASR product found within the crack of an aggregate [20], where silicate is abundant while little calcium can be expected. Actually, considering the surface of the alkali silicate slurry in the system of S1 not being completely covered by the added calcium shown in Figure 5, the free transport of alkali silicate can be expected to ensure the availability of silicate to react with the added calcium as much as possible.

As shown in Figure 12a for Sample S2 with a Ca(OH)_2_ paste addition of 5 g, most of the areas near the border of the hard layer with the adhesives at the top had high values of the Ca/Si ratio ranging from 0.40–0.60, except for two areas with the Ca/Si ratio of around 0.72 and 2.15, which are indicated with an arrow in Figure 12a. The areas from the second and the third rows of indents had a lower Ca/Si ratio ranging from 0.20–0.40. This decreasing trend of the Ca/Si ratio when getting away from the border of the hard layer and the adhesives was caused by the presence of Ca(OH)_2_ in the region of the adhesives. The location of this Ca(OH)_2_ was expected to be near the area with a Ca/Si ratio of 2.15, since calcium alkali silicate alone cannot give such a high value of Ca/Si ratio [21]. Clearly, the presence of additional Ca(OH)_2_ near the surface of the hard layer can increase the amount of calcium available for its interaction with alkali silicate to form a calcium alkali silicate with a high Ca/Si ratio. This is the reason for the areas from the first row showing a higher Ca/Si ratio than the others. Notably, the influence of this effect faded as the areas got further away from the adhesives containing Ca(OH)_2_, resulting in the decreased Ca/Si ratio of the areas in the second and the third rows. The location of the invalid indent is marked with a circle.

As shown in Figure 12b, most of the areas (93%) had a Ca/Si ratio ranging from 0.30–0.50, and 83% of the areas had a (Na + K)/Si ratio ranging from 0.30–0.40. Comparing with the results of S1, clearly the areas from S2 had generally a higher Ca/Si ratio resulting from the addition of more calcium or, more specifically, the presence of Ca(OH)_2_ in the adhesives.

The Ca/Si ratio variation of the indented areas from Sample S3 is shown in Figure 13a. Generally speaking, the distribution of the Ca/Si ratio of the indented areas did not show any typical pattern except several “islands” with a higher Ca/Si ratio, as well as the areas near the border of the hard layer and the adhesives having a lower Ca/Si ratio. Notably, the location of the area indicated with “G” in Figure 13a having a higher Ca/Si ratio than its neighbors is consistent with the one indicated with “G” in Figure 10 having a higher elastic modulus and higher grey scale level than its neighbors. This indicates that both a higher Ca/Si ratio and a more densified structure in this region contributed to the higher elastic modulus of this region. The location of the invalid indent is marked with a circle.

According to Figure 13b, most of the analyzed areas (84%) had a Ca/Si ratio ranging from 0.20–0.30; some areas had a higher Ca/Si ratio ranging from 0.30–0.40; while one area had a Ca/Si ratio of about 0.44; 84% of the areas had a (Na + K)/Si ratio varying from 0.30–0.40.

Comparing all of the results with the ones of Brouxel [22], the calcium alkali silicate constituting the hard layer from S1 in this study had a similar Ca/Si ratio and (Na + K)/Si ratio as the reaction rim, which was located at about 50 μm from the aggregate surface; the calcium alkali silicate constituting the hard layer from S2 had a similar Ca/Si ratio and (Na + K)/Si ratio as the reaction rim, which was located at about 80 μm from the aggregate surface; the calcium alkali silicate constituting the hard layer from S3 had a similar Ca/Si ratio and (Na + K)/Si ratio as the reaction rim, which was located at about 60 μm from the aggregate surface. Besides, for the ASR gel [23], the one formed at the place closer to the cement paste than the aggregate has a higher Ca/Si ratio; the one formed at the place closer to the aggregate than the cement paste has a lower Ca/Si ratio. Therefore, presumably, the calcium alkali silicate from S1 can be considered as the product formed close to the aggregate surface; the calcium alkali silicate from S2 can be considered as the product formed close to the cement paste; the calcium alkali silicate from S3 can be considered as the product formed at an intermediate place between the former ones.

Regarding the situation in S2 and S3, notably, the hard layer from S2 (less calcium was added) was composed of calcium alkali silicate with a higher Ca/Si ratio; while the hard layer from S3 (more calcium was added) was composed of calcium alkali silicate with a lower Ca/Si ratio. However, this cannot be considered as contradictory, since the region from S2 was located close to the Ca(OH)_2_ paste, where Ca(OH)_2_ was abundantly present, while the region from S3 was located close to the alkali silicate slurry, where little Ca(OH)_2_ was present. Therefore, the Ca/Si ratio of the hard layer was more influenced by its location after the complete coverage of the alkali silicate slurry, i.e., a higher Ca/Si ratio when it is close to the calcium and a lower Ca/Si ratio when it is close to the alkali silicate.

### 3.3. Elastic Modulus vs. Elemental Composition

**Figure 14:**
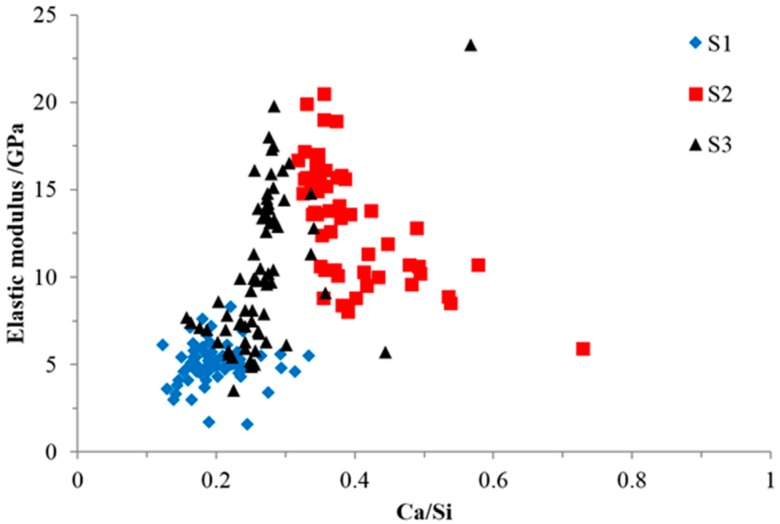
The relationship between elastic modulus and Ca/Si ratio.

The elastic modulus and Ca/Si ratio of all of the indented areas are plotted in Figure 14 to investigate their relationship.

As shown in Figure 14, the general relationship between the elastic modulus and the Ca/Si ratio of the indented areas from all of the samples was consistent with the previous research [18,24] stating that the elastic modulus increased with the increase of the Ca/Si ratio. This was due to the formation of a more complex and interlinked silica network of calcium alkali silicate [25] encouraged by an increased amount of calcium. In S1, calcium was too sparse to ensure its continuous interaction with alkali silicate to form the calcium alkali silicate with a high Ca/Si ratio and elastic modulus, especially given that alkali silicate was sufficiently supplied through the uncovered surface of the slurry layer (shown in Figure 5) for its interaction with existing calcium alkali silicate, as well [19]. In S2, due to the greater presence of Ca(OH)_2_ in the adhesives attached to the hard layer, more calcium was available to be incorporated into the silicate network and to form a calcium alkali silicate with a higher Ca/Si ratio and elastic modulus.

However, it should be noted that the influence of the Ca/Si ratio on the elastic modulus is different from sample to sample. Therefore, ANCOVA analysis was performed to evaluate the influence of other factors, e.g., porosity, on the relationship between the Ca/Si ratio and the elastic modulus within each sample and for all of the samples. A level of significance of 0.05 was used. The results of the analysis for all of the samples show that the amount of diversity of the elastic modulus determined by the Ca/Si ratio is 62.7%; the rest is determined by other factors, including porosity. For the analysis of each sample, the amount of diversity of the elastic modulus determined by the Ca/Si ratio in S1 is 0.5%. For S2, the amount of diversity of the elastic modulus determined by the Ca/Si ratio is 46.4%, while for S3, it is 27.7%. This indicates that the influence of other factors on the relationship between the elastic modulus and the Ca/Si ratio is the highest in S1 and the lowest in S2. This featured phenomenon can be explained as follows.

As shown in Figure 9b and Figure 12b for S2, the areas with the Ca/Si ratio higher than 0.40 were located near the interface of the hard layer with its adhesives, where a porous and weak structure was expected. Hence, the elastic moduli of these areas were low even though the calcium alkali silicate here had a higher value of the Ca/Si ratio. Simply stated, it was the porosity that predominantly determined the elastic modulus in this region rather than the Ca/Si ratio. At the same time, as shown in Figure 10b and Figure 13b for S3, the areas with higher Ca/Si ratio of about 0.30 having a densified structure were located far away from the border of the hard layer with its adhesives. Hence, these areas had higher elastic moduli resulting from the higher Ca/Si ratio and the lower local porosity. Moreover, the relatively concentrated points from S1 in Figure 14 implied that the pore distribution is uniform and that a similar porous structure can be speculated in the whole region, given a concentrated distribution of the Ca/Si ratio.

It should be noted that the variation between the depth of indentation and the interaction volume of SEM [26] could influence the results of this study. Besides, the impregnation of epoxy filling the capillary pores of the samples during the preparation of the samples for nano-indentation and SEM-EDS analysis could also have some influence. However, without impregnation, it would be very difficult to investigate the mechanical properties of the samples obtained from the simulated calcium-alkali-silicate system in this study.

### 3.4. Discussion of the Elastic Modulus of the Hard Layer and Expansive Pressure in ASR

As stated in Section 3.2, the calcium alkali silicate constituting the hard layers from the three systems can represent the calcium alkali silicate formed at different places, where different amounts of calcium are available for interaction with alkali silicate resulting in the different Ca/Si ratios of the formed calcium alkali silicate: the calcium alkali silicate with a low Ca/Si ratio obtained from S1 can be considered to be formed close to the aggregate surface, where little calcium, while abundant alkali silicate are present; the calcium alkali silicate with a high Ca/Si ratio obtained from S2 can be considered to be formed close to the cement paste, where abundant calcium, while little alkali silicate are present; the calcium alkali silicate with a moderate Ca/Si ratio obtained from S3 can be considered to be formed at an intermediate place.

**Figure 15:**
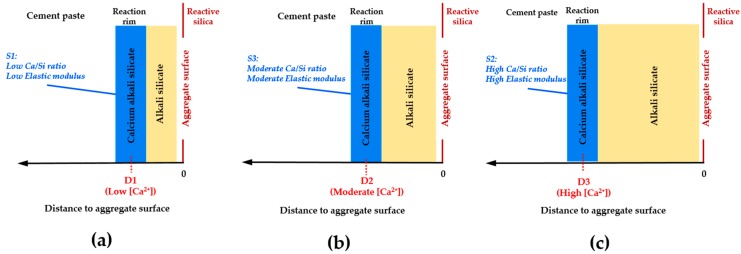
Schematic representation of the reaction rim formed at different locations (D1 < D2 < D3). (**a**) The reaction rim formed at a place close to the aggregate surface; (**b**) the reaction rim formed at a place with a moderate distance to either the cement paste or the aggregate surface; (**c**) the reaction rim formed at a place close to the cement paste.

**Figure 16:**
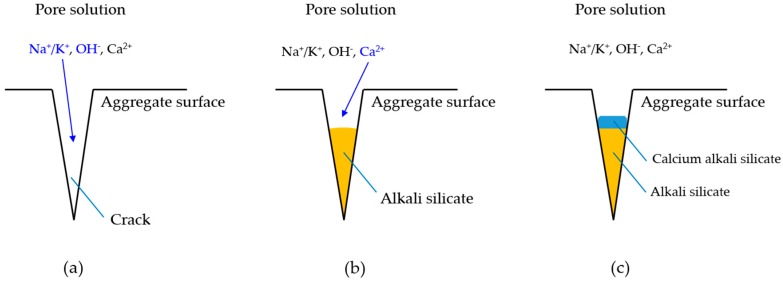
Schematic representation of situation in the interior of a crack in an aggregate. (**a**) Attack of alkalis and hydroxyls on the reactive silica in a crack; (**b**) formation of alkali silicate and its subsequent interaction with calcium; (**c**) formation of calcium alkali silicate.

Based on the results of the present study, the calcium alkali silicate with a low Ca/Si ratio from S1 had a low value of the elastic modulus. This implies that this kind of calcium alkali silicate can make up the reaction rim, which is formed close to the aggregate surface where little alkali silicate is present, as shown in Figure 15a. Alternatively, the calcium alkali silicate with a high Ca/Si ratio from S2 had a high value of the elastic modulus. This implies that this kind of calcium alkali silicate can make up the reaction rim, which is formed close to the cement paste when a large amount of alkali silicate is present, as shown in Figure 15c. Furthermore, the calcium alkali silicate with a moderate Ca/Si ratio from S3 had a moderate value of the elastic modulus, implying the formation of the reaction rim at an intermediate position compared to the former situations shown in Figure 15b. This provides evidence to explain why the damage caused by ASR often happens around the aggregate rather than in the hardened cement paste. Notably, for the situation where the damage of ASR happens within the aggregate, the reaction rim (the hard layer in this study) can form at the opening of the crack to the cement paste and behave similarly. This means that the alkali silicate present in the interior of the crack interacts with the calcium coming from the pore solution of the cement paste to form calcium alkali silicate blocking the opening of the crack to the cement paste, as shown in Figure 16a–c.

### 3.5. Further Discussion

**Figure 17:**
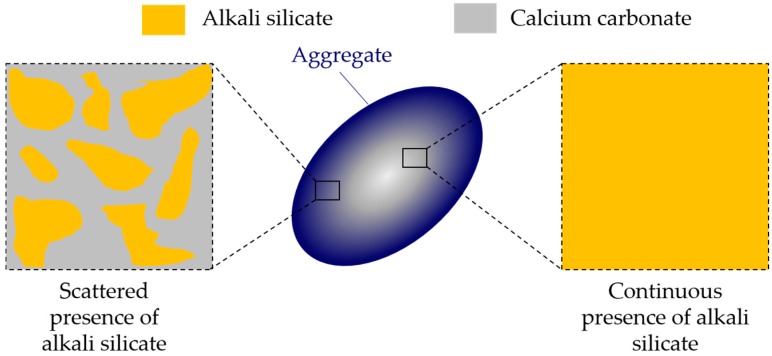
Schematic representation of the different presences of alkali silicate.

In the chemical model system of this study, pore solution is assumed to be the only source of calcium, consistent with the situation for aggregates like opal, chert and sandstone, where little calcium is present around the reactive silica in the aggregates. This leads to the formation of a continuous phase of alkali silicate, as shown in Figure 4b. For the aggregates coming from calcareous rock, calcium is abundantly contained in the calcium carbonate surrounding the reactive silica. However, this kind of calcium cannot be considered as active as the one provided by the pore solution. Because calcium carbonate has an extremely low solubility in water at room temperature (about 13 mg/L, K_sp_ = 3.3 × 10^−9^) compared to calcium hydroxide with its solubility at about 1.7 g/L (K_sp_ = 5.5 × 10^−6^), therefore, this kind of calcium is actually “locked” in the calcium carbonate that surrounds the reactive silica in the calcium-rich aggregates. Accordingly, the calcium carbonate can be considered as an inert phase, which separates or even isolates the reactive silica in aggregates. In this sense, a continuous phase of alkali silicate shown in Figure 4b and Figure 17 will be altered into various regions of alkali silicate separated by the calcium carbonate phase, as shown in Figure 17. Notably, each region of alkali silicate can still be considered as a continuous phase of alkali silicate at a smaller scale.

Bearing in mind that the findings in the present study are based on the chemical model system idealizing multiple physico-chemical reactions of ASR, directly applying those findings to real concrete might be misleading.

## 4. Conclusions

After chemically realizing the formation of the reaction rim at the interface of alkali silicate and Ca(OH)_2_ in a chemical model in our prior work, the elastic modulus of the calcium alkali silicate constituting this idealized reaction rim was investigated in this study.

The elastic modulus of the calcium alkali silicate constituting the reaction rim (the hard layer in this study) varied from 5 to 20 GPa. The elastic modulus increased with the increase of the Ca/Si ratio. Furthermore, the more calcium was available for its interaction with alkali silicate to form calcium alkali silicate, the higher the Ca/Si ratio was and, consequently, the higher the elastic modulus of the formed calcium alkali silicate.

Our original results will benefit a better understanding about the mechanical properties of the calcium alkali silicate formed at the interface between alkali silicate and Ca(OH)_2_ in a chemical model of ASR. Any extrapolation of these findings to real concrete should be critically conducted. Further research will focus on the dynamic mechanism between the build-up of the expansive pressure of ASR and the constraint capacity offered by the calcium alkali silicate from the reaction rim.

## Figures and Tables

**Table 1:** Chemical composition of the used silica fume/wt %.

Compositions	SiO_2_	CaO	Al_2_O_3_	Fe_2_O_3_	MgO	Na_2_O	K_2_O	SO_3_
Content	94.2	0.6	1.0	0.5	0.7	1.0	1.1	0.3
